# Prevalence and risk factors associated with lymphatic filariasis in American Samoa after mass drug administration

**DOI:** 10.1186/s41182-017-0063-8

**Published:** 2017-08-04

**Authors:** Shaun P. Coutts, Jonathan D. King, Molisamoa Pa’au, Saipale Fuimaono, Joseph Roth, Mary Rose King, Patrick J. Lammie, Colleen L. Lau, Patricia M. Graves

**Affiliations:** 1 College of Public Health, Medical and Veterinary Sciences, James Cook University, Cairns, Australia; 2 Department of Control of Neglected Tropical Diseases, World Health Organization, Geneva, Switzerland; 3 American Samoa Department of Health, Pago Pago, American Samoa; 4 Office of Public Health Preparedness and Response, Centers for Disease Control and Prevention, Atlanta, USA; 5 Independent Nursing Consultant, Geneva, Switzerland; 6 Neglected Tropical Diseases Support Center, The Task Force for Global Health, Atlanta, USA; 7 Department of Global Health, Research School of Population Health, Australian National University, Canberra, Australia; 8 Australian Institute of Tropical Health and Medicine and College of Public Health, Medical and Veterinary Sciences, James Cook University, Cairns, Australia

**Keywords:** Lymphatic filariasis, American Samoa, Prevalence, MDA, Epidemiology, *Wuchereria bancrofti*, PacELF

## Abstract

**Background:**

In 2000, American Samoa had 16.5% prevalence of lymphatic filariasis (LF) antigenemia. Annual mass drug administration (MDA) was conducted using single-dose albendazole plus diethylcarbamazine from 2000 to 2006. This study presents the results of a 2007 population-based PacELF C-survey in all ages and compares the adult filarial antigenemia results of this survey to those of a subsequent 2010 survey in adults with the aim of improving understanding of LF transmission after MDA.

**Results:**

The 2007 C-survey used simple random sampling of households from a geolocated list. In 2007, the overall LF antigen prevalence by immunochromatographic card test (ICT) for all ages was 2.29% (95% CI 1.66–3.07). Microfilaremia prevalence was 0.27% (95% CI 0.09–0.62). Increasing age (OR 1.04 per year, 95% CI 1.02–1.05) was significantly associated with ICT positivity on multivariate analysis, while having ever taking MDA was protective (OR 0.39, 95% CI 0.16–0.96). The 2010 survey used a similar spatial sampling design.

The overall adult filarial antigenemia prevalence remained relatively stable between the surveys at 3.32% (95% CI 2.44–4.51) by ICT in 2007 and 3.23 (95% CI 2.21–4.69) by Og4C3 antigen in 2010. However, there were changes in village-level prevalence. Eight village/village groupings had antigen-positive individuals identified in 2007 but not in 2010, while three villages/village groupings that had no antigen-positive individuals identified in 2007 had positive individuals identified in 2010.

**Conclusions:**

After 7 years of MDA, with four rounds achieving effective coverage, a representative household survey in 2007 showed a decline in prevalence from 16.5 to 2.3% in all ages. However, lack of further decline in adult prevalence by 2010 and fluctuation at the village level showed that overall antigenemia prevalence at a broader scale may not provide an accurate reflection of ongoing transmission at the village level.

## Background

Lymphatic filariasis (LF) is a neglected mosquito-borne parasitic disease caused by three species of filarial parasites—*Wuchereria bancrofti*, *Brugia malayi* and *Brugia timori* [1]. In May 1997, the World Health Assembly passed a resolution calling for the global elimination of LF as a public health problem by the year 2020. The World Health Organisation (WHO) subsequently launched the Global Programme to Eliminate Lymphatic Filariasis (GPELF) in 2000 to facilitate this aim [2]. In 1999, the Pacific Programme for the Elimination of Lymphatic Filariasis (PacELF) was formed to coordinate regional efforts toward elimination in the 22 Pacific Island countries and territories (PICTs) by 2020, utilising a strategy of annual mass drug administration (MDA) of a single dose of diethylcarbamazine (DEC) plus albendazole for the entire at-risk population [3].

The WHO criteria for ceasing MDA after a minimum of five effective annual rounds (coverage exceeding 65% of the total population) in areas where *Aedes* mosquitos are the primary vector is <1% antigenemia in a transmission assessment survey (TAS) of 6- to 7-year-old children. Critical cut-off values are calculated based on sample sizes designed so that a TAS evaluation unit (EU) has at least a 75% chance of passing if antigenemia is 0.5% and no more than 5% chance of passing if antigenemia is ≥1% [4]. Prior to the development of the TAS, PacELF had established separate criteria for ceasing MDA if <1% filarial antigenemia (<2% upper 95% CI) across all age groups, based on the results of a population-based cluster survey (C-survey).

American Samoa is an unincorporated territory of the USA. The population of 54,454 (in 2015) inhabits the islands of Tutuila, Aunu’u, Ofu-Olosega and Ta’u. The capital, Pago Pago, is located on Tutuila, the largest island, where >95% of the population reside. The territory was partitioned from neighbouring Samoa in 1899: however, the two communities continue to share strong family, cultural, linguistic and economic bonds. LF in American Samoa is caused by the diurnally sub-periodic *Wuchereria bancrofti*, transmitted predominantly by the day-biting mosquito *Aedes polynesiensis*, with the night-biting *Aedes samoanus* as a secondary vector [5–7].

LF prevalence surveys have been conducted in American Samoa since 1923, with microfilaremia (Mf) prevalence in pre-PacELF surveys as high as 21% in 1962 (*n* = 1000). MDA with DEC (6 mg/kg monthly for a total of 72 mg/kg over a year) was undertaken in 1963 and 1965. A follow-up survey in 1968 found Mf prevalence had decreased to 0.3% (*n* = 1053 across 13 villages) [3]. No further MDA interventions were recorded until the commencement of PacELF and the national elimination programme in 1999, when a nationwide convenience survey of 18 villages established a baseline prevalence of 16.5% (*n* = 3018) filarial antigenemia by immunochromatographic card test (ICT) [3]. Seven rounds of annual MDA followed during 2000–2006, targeting the whole population except pregnant women, children less than 2 years old and the severely ill [8].

MDA coverage in American Samoa was initially poor, ranging from 19% in 2000 to 49% in 2002. From 2002 to 2005, the national programme was independently evaluated using a variety of formative research methods including focus groups with drug distributors (programme directors, nurses, health assistants and volunteers); a multi-stage household cluster survey of community knowledge, attitudes and practices; and key informant interviews with church leaders. The programme evaluation resulted in significant changes to community mobilisation, behaviour change communication and drug delivery strategies. Notably, the evaluation identified the involvement of churches as a key driver of improved programme coverage, with over half of the population receiving treatment in conjunction with church attendance [9]. Coverage increased to 71% in 2003 and was sustained at a relatively high level in 2004 (65%), 2005 (67%) and 2006 (70%) [9].

A significant decrease in LF antigen prevalence was observed in sentinel villages between 2001 and 2006, coinciding with the improvements in MDA participation and coverage [10]. Two spot check village surveys in 2006 (a total of four villages) found higher prevalence outside of sentinel villages [8, 10, 11].

This paper reports on the results of a 2007 population-based PacELF C-survey and compares the proportion of antigen-positive adults in 2007 with those found in a 2010 study for corresponding villages. We aimed to identify potential risk factors for infection in 2007 and examine small-scale changes in antigenemia over time by comparing village-level adult seroprevalence between the 2007 and 2010 surveys. Furthermore, we aimed to reflect on how these small-scale variations in disease transmission may affect post-MDA surveillance practices and strategies.

## Methods

### Data sources

The 2007 C-survey data were collected by American Samoa Department of Health (AS DOH) staff with the support of staff from the US Centers for Disease Control and Prevention (US CDC). The survey team used geographic information systems (GIS) data from the American Samoa Department of Commerce to identify buildings in villages on the populated islands of Tutuila, Aunu’u, Ofu-Olosega and Tau. Under the assumption of an average household size of five individuals, a simple random sample of 540 buildings was taken (500 in Tutuila and Aunu’u, 20 in Ofu-Olosega, 20 in Tau), with a target sample size of 2700 individuals. GIS software (ArcGIS, Environmental Systems Research Institute, Redlands, CA) was used to identify and highlight selected buildings and generate printed maps for use by survey teams to identify these households on-ground. All individuals 2 years of age and older residing in selected households were invited to participate and tested for filarial antigenemia using ICT (BinaxNOW Filariasis ICT Alere, Scarborough, USA). ICT-positive individuals were tested for Mf by microscopic examination of thick blood smears (20 μL of blood, Giemsa-stained) and treated with DEC and albendazole [12]. Where possible, those who were absent at the time of visit were tracked down and tested according to the same procedure. A standard questionnaire was administered at the time of testing to collect data on demographics and MDA compliance history. All individuals involved in the survey provided verbal consent prior to participation.

The 2010 survey data were collected as part of a leptospirosis seroprevalence study, using a spatially representative household sampling design similar to the C-survey. The study was designed to include a representative sample of the adult population (≥18 years old), and methods and sampling designs have been previously described [13, 14]. The serum bank was subsequently used for a study of the seroprevalence and spatial epidemiology of LF in American Samoa after MDA, which identified possible residual foci of antigen-positive adults [15]. Filarial antigenemia in stored venous serum samples was measured through the detection of circulating filarial antigen (CFA) using the Og4C3 antigen ELISA test (cut-off value of >32 units was considered seropositive).

### Data analysis

The 2007 survey data were entered into Microsoft Excel before being exported into STATA 13.1 (College Station, TX), which was used for subsequent analyses. Descriptive statistics were derived from the data to examine the characteristics of the sample. Univariate logistic regression analysis was performed on independent variables (sex, age, years lived in American Samoa, having ever taken MDA, number of years having taken MDA, island of residence) to assess association with the dependent variable (ICT positivity). Years lived in American Samoa was categorised into <7 and ≥7 years in order to assess possible differences in risk between individuals who had lived in American Samoa prior to MDA and those who had lived in American Samoa only during MDA (<7 years). All independent variables with a *P* value of <0.25 on univariate analysis were considered for inclusion in a full multivariate logistic regression model. Starting with all potentially significant independent variables from the univariate analysis, a backward stepwise regression procedure (*P* ≤ 0.05) was performed to refine and select the final set of variables for inclusion in the multivariate model.

For comparison of village-level adult filarial antigenemia prevalence between the 2007 and 2010 surveys, villages in which five or more individuals were sampled in each survey were included for analysis. Some small adjacent villages that did not meet this cut-off were combined into village groupings for this analysis if the groupings were considered to be ecologically and geographically appropriate. Villages which could not be grouped and did not meet the cut-off were excluded from the comparison. As the 2010 survey only sampled adults, individuals under 18 years of age were excluded from the 2007 data for the purposes of the comparison.

## Results

### 2007 survey

From a total of 540 buildings selected, 2216 individuals from 394 buildings were identified as being eligible for inclusion in the survey. Of these individuals, 1881 were available for testing and enrolled in the survey. The most common reasons for an individual being missed by the survey (*n* = 335) were not being home at the time (59.9%), followed by being at work (22.3%), being off-island (13.3%) and refusing to participate (4.6%). The sample population is described in Table 1.

**Table 1:** Characteristics of the eligible sampled and missed population in the 2007 lymphatic filariasis C-survey in American Samoa

	Eligible (%)	Sampled (%)	Missed (%)
Total	2216	1881	335
Gender			
Female	1150 (51.9)	1000 (53.2)	150 (48.8)
Male	1066 (48.1)	881 (46.8)	185 (52.2)
Age group (years)			
2–9	293 (13.2)	269 (14.3)	24 (7.2)
10–19	601 (27.1)	512 (27.2)	89 (26.6)
20–29	325 (14.7)	266 (14.1)	59 (17.6)
30–39	330 (14.9)	273 (14.5)	57 (17.0)
40–49	296 (13.4)	235 (12.5)	61 (18.2)
50–59	186 (8.4)	169 (9.0)	17 (5.1)
60 +	185 (8.3)	157 (8.3)	28 (8.4)
Island of residence			
Tutuila/Aunu’u	2094 (94.5)	1773 (94.3)	321 (95.8)
Ofu-Olosega	57 (2.6)	53 (2.8)	4 (1.2)
Ta’u	65 (2.9)	55 (2.9)	10 (3.0)

**Fig. 1:**
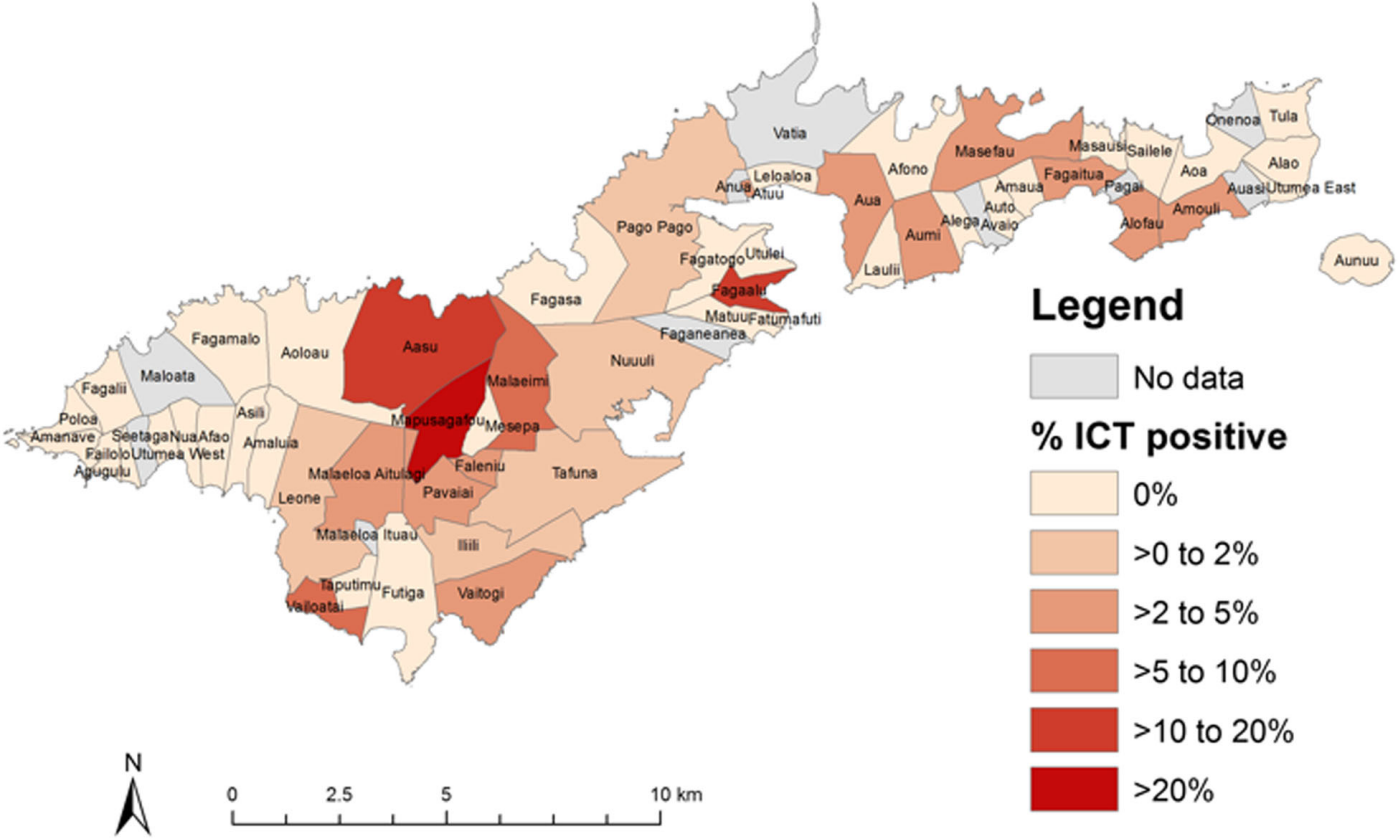
Village-level filarial antigenemia prevalence in the 2007 lymphatic filariasis C-survey in American Samoa—Tutuila and Aunu’u

**Fig. 2:**
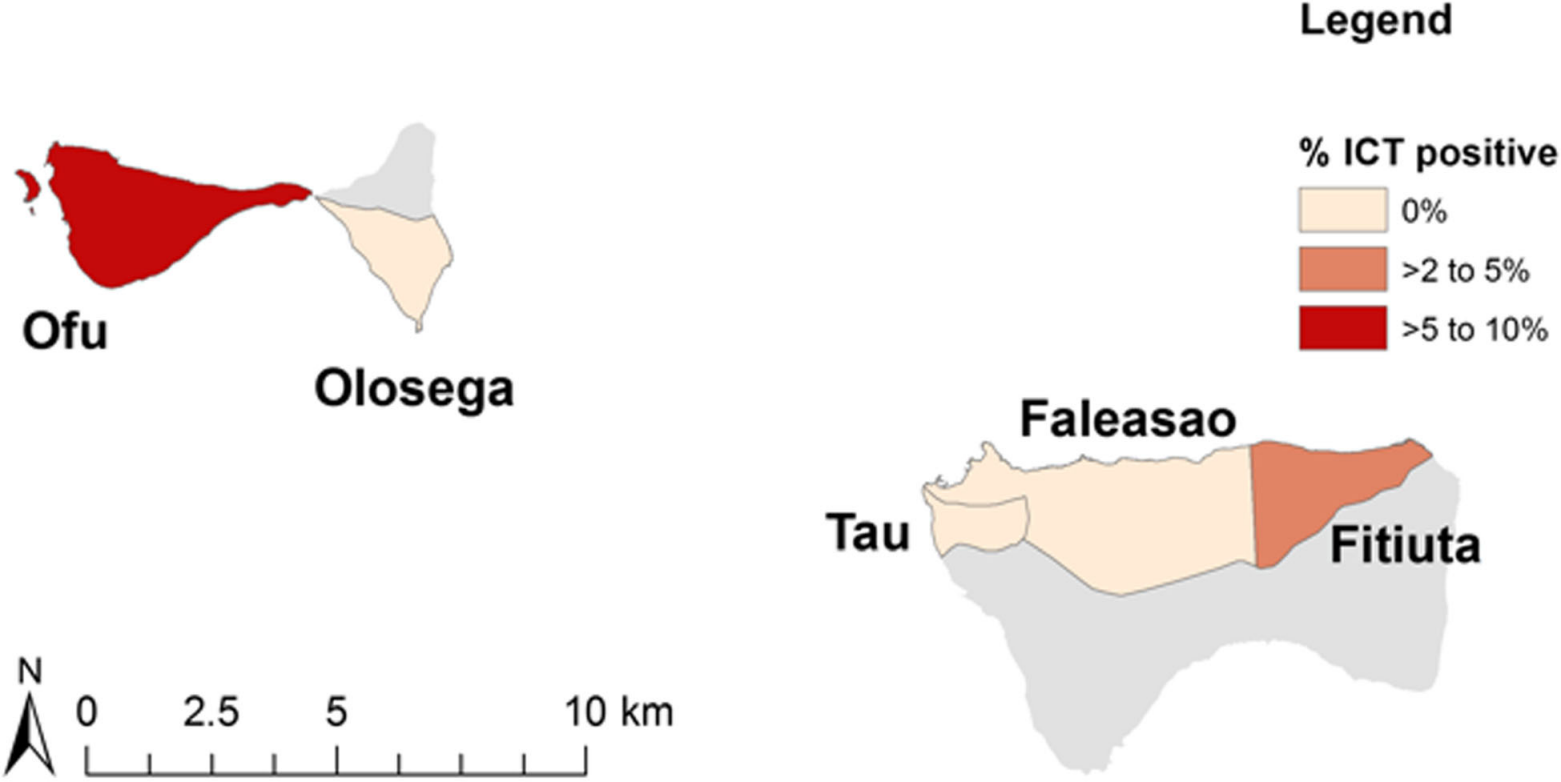
2007 village-level filarial antigenemia prevalence in the 2007 lymphatic filariasis C-survey in American Samoa—Ofu-Olosega and Tau

**Fig. 3:**
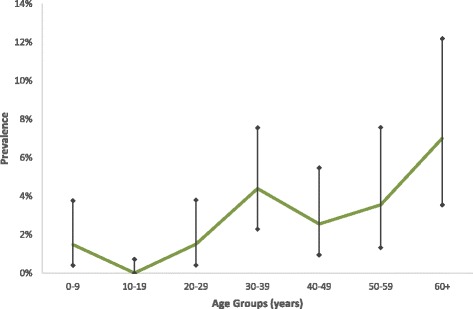
Age-specific prevalence for *W. bancrofti* antigenemia in the 2007 lymphatic filariasis C-survey in American Samoa. Bars indicate 95% confidence intervals

The overall LF antigenemia prevalence for all ages by ICT was 2.29% (43/1881, 95% CI 1.66–3.07). Table 2 summarises LF antigenemia prevalence by island group. Variations in village-level LF antigenemia prevalence are described in Figs. 1 and 2. Four ICT-positive children aged <10 years were identified, all residents on the island of Tutuila. Age-specific LF antigenemia prevalence is shown in Fig. 3.

**Table 2:** *W. bancrofti* antigenemia prevalence by island group in the 2007 lymphatic filariasis C-survey in American Samoa

Island	No. of participants	No. of ICT positive	Prevalence (%)
Tutuila/Aunu’u	1773	40	2.26
Ofu-Olosega	53	2	3.77
Ta’u	55	1	1.82

Microfilaremia prevalence was 0.27% (5/1881, 95% CI 0.09–0.62). Of the five microfilaremic individuals identified, all resided in different villages on the island of Tutuila. Three were aged 30–39 and two were aged ≥60 years.

A number of independent variables were significantly associated with being ICT positive (*P* < 0.25) on univariate logistic regression analysis and included in the full multivariate model. Table 3 provides a summary of associations between independent variables and ICT positivity on univariate logistic regression analysis.

**Table 3:** Association between variables and immunochromatographic card test (ICT) positivity on univariate logistic regression analysis for the 2007 lymphatic filariasis C-survey in American Samoa

	No. of participants	ICT positive	Prevalence (%)	Univariate OR (95% CI)	*P* value
Gender					
Female	1000	18	1.80	Reference	
Male	881	25	2.84	*1.59 (0.86–2.94)*	*0.14*
Age					
1-year increments	1881	43	2.29	*1.04 (1.02–1.05)*	*<0.001*
Years lived in AS					
<7 years	303	9	2.97	1.39 (0.66–2.92)	0.39
≥7 years	1575	34	2.16	Reference	
Ever taken MDA					
No	109	6	5.50	Reference	
Yes	1763	37	2.10	*0.37 (0.15–0.89)*	*0.03*
Years taken MDA					
0	115	6	5.22	Reference	
1	134	6	4.48	0.85 (0.27–2.72)	0.79
2	183	3	1.64	*0.3 (0.07–1.24)*	*0.10*
3	218	2	0.92	*0.17 (0.03–0.85)*	*0.03*
4	256	7	2.73	*0.51 (0.17–1.55)*	*0.24*
5	647	13	2.01	*0.37 (0.14–1.00)*	*0.05*
6	299	6	2.01	*0.37 (0.12–1.18)*	*0.09*
7	9	0	0.00	–	–
Island of residence					
Tutuila	1773	40	2.26	Reference	
Ofu-Olosega	53	2	3.77	1.7 (0.4–7.22)	0.47
Ta’u	55	1	1.82	0.8 (0.11–5.94)	0.83

Italicized type indicates significance for inclusion in the full multivariate model

Increasing age (OR 1.04 per year, 95% CI 1.02–1.05) remained significantly associated with ICT positivity in the final multivariate logistic regression model, while having ever taken part in an MDA round was protective (OR 0.39, 95% CI 0.16–0.96). Table 4 provides a summary of the final multivariate logistic regression model.

**Table 4:** Variables significantly associated with immunochromatographic card test (ICT) positivity on multivariate logistic regression analysis in the 2007 lymphatic filariasis C-survey in American Samoa

Variable	Adjusted odds ratio	95% confidence interval	*P* value
Age (1-year increments)	1.04	1.02–1.05	<0.001
Ever taken MDA	0.39	0.16–0.96	0.04

### Adult filarial antigenemia—comparison between the 2007 and 2010 surveys

The adult filarial antigenemia was 3.32% (95% CI 2.44–4.51) by ICT in 2007 and 3.23% (95% CI 2.21–4.69) by Og4C3 in 2010. The overall adult filarial antigenemia prevalence remained relatively stable between the surveys.

Forty-four villages were included for village-level comparison, consisting of 23 individual villages and eight village groupings (of between two to four villages). Antigen-positive individuals were identified in both the 2007 and 2010 surveys in nine (29.0%) of the 31 villages/village groupings. Eight (25.8%) village/village groupings had antigen-positive individuals identified in 2007 but not in 2010, while three (9.6%) villages/village groupings that had no antigen-positive individuals identified in 2007 had positive individuals identified in 2010. Table 5 describes village-level filarial antigenemia in the 2007 and 2010 surveys.

**Table 5:** Village-level adult filarial antigenemia prevalence in the 2007 and 2010 lymphatic filariasis surveys in American Samoa

Village/village grouping	2007 Tested	2007 ICT positive	2007 % positive	95% CI	2010 Tested	Og4C3 Positive	2010 % positive	95% CI
Aasu Fou/Aoloau Fou	22	1	4.55	0.12–22.84	35	1	2.86	0.07–4.92
Afao/Amaluia/Asili	7	0	0	0–40.96	50	2	4.00	0.49–13.71
Agugulu/Nua/Seetaga	29	0	0	0–11.94	28	0	0	0–12.34
Alao	13	0	0	0–24.71	19	0	0	0–17.65
Alega/Amaua/Auto/Avaio	21	0	0	0–16.11	23	2	8.70	1.07–28.04
Amouli	17	1	5.88	0.15–28.69	18	1	5.56	0.14–27.29
Aoa	7	0	0	0–40.96	15	0	0	0–21.80
Aua	39	3	7.69	1.62–20.87	22	1	4.55	0.12–22.84
Auasi/Utumea East	12	0	0	0–26.46	13	0	0	0–24.71
Aumi/Laulii	22	1	4.55	0.12–22.84	23	0	0	0–14.82
Aunuu	10	0	0	0–30.85	16	0	0	0–20.59
Fagaalu	22	3	13.64	2.91–34.91	17	0	0	0–19.51
Fagaitua/Pagai	13	1	7.69	0.19–36.03	19	0	0	0–17.65
Fagali’i	8	0	0	0–36.94	13	4	30.77	9.09–61.43
Fagasa	10	0	0	0–30.85	17	0	0	0–19.51
Fagatogo	27	0	0	0–12.77	21	0	0	0–16.11
Faleasao/Fitiuta/Tau	31	1	3.23	0.08–16.70	43	0	0	0–8.22
Faleniu	22	2	9.09	1.12–29.16	18	0	0	0–18.53
*Ili’ili*	*39*	*1*	*2.56*	*0.06–13.48*	*23*	*4*	*17.39*	*4.95–38.78*
Leone	89	1	1.12	0.03–6.10	20	0	0	0–16.84
Malaeimi	39	2	5.13	0.77–20.81	28	1	3.57	0.09–18.35
Malaeloa	17	1	5.88	0.15–28.69	30	3	10.00	2.11–26.53
Mesepa	16	0	0	0–20.59	7	0	0	0–40.96
Ofu	19	2	10.53	1.30–33.14	11	0	0	0–28.49
Olosega	10	0	0	0–30.85	14	0	0	0–23.16
Pago Pago	88	2	2.27	0.28–7.97	73	2	2.74	0.33–9.55
Pavaiai	70	2	2.86	0.35–9.94	16	1	6.25	0.16–30.23
Tafuna	135	3	2.22	0.46–6.36	21	0	0	0–16.11
Taputimu	10	0	0	0–30.85	14	0	0	0–23.16
Tula	7	0	0	0–40.96	26	0	0	0–13.23
Vaitogi	30	2	6.67	0.82–22.07	7	1	14.29	0.36–57.87

Italicized type indicates statistical significance at the .05 level

**Fig. 4:**
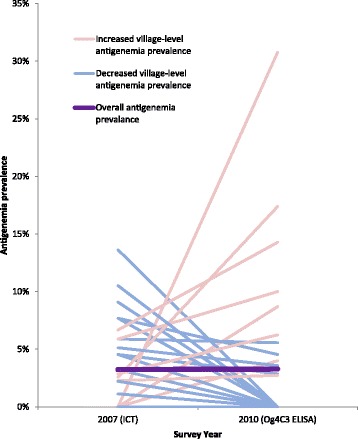
Comparison of overall and village-level adult filarial antigenemia prevalence between the 2007 and 2010 lymphatic filariasis surveys in American Samoa

The largest discrepancies in proportions of antigen-positive adults between 2007 and 2010 were in the villages of Fagali’i (0 to 33.77%, *P* = 0.08), Ili’ili (2.56 to 17.39%, *P* = 0.04), Faga’alu (13.64 to 0%, *P* = 0.11) and Ofu (10.53 to 0%, *P* = 0.27). Figure 4 illustrates the changes in filarial antigenemia prevalence in villages/village groupings between 2007 and 2010.

## Discussion

Since the commencement of PacELF in 1999, when a nationwide mapping survey established a baseline prevalence of 16.5% antigenemia, American Samoa has made significant progress toward the elimination of LF. A timeline of LF programme activities in American Samoa is presented in Table 6. Antigenemia declined to 2.29% in all ages by 2007 after four effective rounds of annual MDA; however, overall antigen prevalence in adults stayed relatively constant over the subsequent 3 years (3.32% in 2007 (ICT) and 3.23% in 2010 (Og4C3 antigen)). Despite remaining at an overall low level, village-based data from the two independent surveys suggest fluctuation in antigen prevalence among adults in some villages.

**Table 6:** Timeline of lymphatic filariasis programme activities in American Samoa, 1999–2011

Year	Activity	Survey population size	Age group	Prevalence (% antigenemia, ICT)	MDA coverage (% total population treated)
1999	Nationwide mapping (convenience survey) [3]	3018 (18 villages)	All	16.5	
2000	MDA [9]				19
2001	MDA [9]				51
2001	Sentinel survey [10]	1024 (4 villages)	All	11.52	
2002	MDA [9]				49
2003	Drug distributor evaluation Community KAP survey Programme modifications [9]				
2003	MDA [9]				71
2003	Drug distributor evaluation Church leader survey Programme modifications [9]				
2003	Sentinel survey [10]	917 (4 villages)	All	13.74	
2004	MDA [9]				65
2004	Drug coverage survey [9]				86
2005	MDA [9]				67
2006	MDA [9]				70
2006	Sentinel survey [10]	1371 (4 villages)	All	0.95	
2006	Purposeful survey [11]	569 (3 villages)	>5 years	4.22	
2007	Random household C-survey (spatial sampling design)	1881 (national)	All	2.29	
2010	Random household survey (spatial sampling design) [15]	807 (national)	≥18 years	3.2 (Og4C3 antigen ELISA)	
2011	TAS [18]	949	6–7 years	0.21	

*ELISA* enzyme-linked immunosorbent assay, *ICT* immunochromatographic card test, *KAP* knowledge, attitudes, practices, *MDA* mass drug administration, *TAS* transmission assessment survey

Both the 2007 and 2010 studies showed a broad distribution of a few infected individuals with some clustering within villages, demonstrating that significant geographic heterogeneity can exist even in such a small island setting. This widespread, low-level distribution has also been demonstrated in a 2011 molecular xenomonitoring study [16]. A subsequent study that compared results of the 2010 human seroprevalence study and the 2011 entomology study provided evidence for good concordance between results from serology and molecular xenomonitoring during post-MDA surveillance in American Samoa [17].

The 2007 survey results did not meet the PacELF criterion of filarial antigenemia <1% (upper CI <2%) for ceasing MDA. The PacELF technical working group recommended that American Samoa undertake an additional round of MDA in 2008; however, this was not accomplished because of limited resources. Even in the absence of additional MDA, American Samoa conducted and passed a TAS in 2011, with a school-based survey of 1st and 2nd graders on Tutuila (*n* = 949, approximating to the 6- to 7-year-old age group) identifying two ICT-positive individuals (0.21%), below the critical cut-off value of six individuals. The two ICT-positive individuals attended the same school on Tutuila, but lived in different villages [18]. A subsequent 2010 seroepidemiological study of adults (using samples originally collected for a leptospirosis study) found evidence of spatial clustering of antigen-positive individuals in two villages on Tutuila (Fagali’i and Ili’ili), suggesting possible residual foci of transmission post-MDA. One of the villages included the school where the two antigen-positive children were identified in the 2011 TAS. Analysis of the demographic data indicated that adult males and recent migrants were at higher risk of LF antigenemia and demonstrated antibody positivity. This association suggests adults may serve as the primary reservoirs for continued transmission, despite low prevalence in younger age groups [15].

TAS and the PacELF C-survey are not designed to provide village-level prevalence estimates, but rather estimates of mean prevalence across an evaluation unit of one or more districts, which was a territory-wide estimate in this island setting. The comparisons between surveys confirm that these cross-sectional survey methods may not always provide an accurate reflection of antigenemia at the smaller-scale village level as expected, given the limited number of persons selected per village. Foci of residual transmission may not be identified which could then serve as areas of potential resurgence. Discrepancies in filarial antigenemia prevalence within some villages between 2007 and 2010 may suggest that foci of local transmission have developed in some villages and declined in others over a relatively short time period. However, these discrepancies could also be explained by limitations in the sampling method, sample sizes and tests used for the comparison.

The findings should be considered in light of the study’s limitations. Although both surveys utilised a similar random spatial sampling method, the 2007 study included multiple adults from the majority of households, while the 2010 study only included one person from the majority of households. No adjustment for clustering within households (which would have widened the confidence intervals around the prevalence estimate) was made for the 2007 dataset, although at the observed level of prevalence it would be expected to have had negligible effect. A further limitation is that the 2007 survey used ICT, while in 2010, stored serum samples collected for a different survey were tested for Og4C3 antigen [15]. Both tests measure circulating filarial antigen, but comparison is hampered by lack of a true gold standard. A multi-country comparison of ICT and Og4C3 antigen in multiple field laboratories concluded that although positive concordance was unexpectedly low at individual level, there was no difference in the prevalence estimates given by the two tests at a population level [19]. More recent studies have shown that concordance between the two tests is much greater when using serum/plasma (as done in this study) rather than dried blood spots for Og4C3 antigen testing [20].

## Conclusions

The variability in village-level antigenemia within the 2007 and 2010 surveys suggests that there remains a significant gap in knowledge regarding LF transmission dynamics at small spatial scales. Further research and a greater understanding of these transmission dynamics, their impact on LF elimination efforts and how to accurately assess significant residual infection may become increasingly important as more countries move into the post-MDA phase and continue to work toward elimination. This research should include further longitudinal studies in villages or areas in which increases or a lack of decline in prevalence has been observed.

In American Samoa specifically, programmatic priorities to support the elimination of LF should include ongoing investigation and surveillance of hotspots identified during surveys, targeted testing and treatment of known high-risk groups and the comparison of TAS results in children against prevalence in older age groups.
